# Halide perovskite nanocrystal arrays: Multiplexed synthesis and size-dependent emission

**DOI:** 10.1126/sciadv.abc4959

**Published:** 2020-09-23

**Authors:** Jingshan S. Du, Donghoon Shin, Teodor K. Stanev, Chiara Musumeci, Zhuang Xie, Ziyin Huang, Minliang Lai, Lin Sun, Wenjie Zhou, Nathaniel P. Stern, Vinayak P. Dravid, Chad A. Mirkin

**Affiliations:** 1 Department of Materials Science and Engineering, Northwestern University, Evanston, IL 60208, USA.; 2 International Institute for Nanotechnology, Northwestern University, Evanston, IL 60208, USA.; 3 Department of Physics and Astronomy, Northwestern University, Evanston, IL 60208, USA.; 4 NU*ANCE* Center, Northwestern University, Evanston, IL 60208, USA.; 5 Department of Chemistry, Northwestern University, Evanston, IL 60208, USA.

## Abstract

Halide perovskites have exceptional optoelectronic properties, but a poor understanding of the relationship between crystal dimensions, composition, and properties limits their use in integrated devices. We report a new multiplexed cantilever-free scanning probe method for synthesizing compositionally diverse and size-controlled halide perovskite nanocrystals spanning square centimeter areas. Single-particle photoluminescence studies reveal multiple independent emission modes due to defect-defined band edges with relative intensities that depend on crystal size at a fixed composition. Smaller particles, but ones with dimensions that exceed the quantum confinement regime, exhibit blue-shifted emission due to reabsorption of higher-energy modes. Six different halide perovskites have been synthesized, including a layered Ruddlesden-Popper phase, and the method has been used to prepare functional solar cells based on single nanocrystals. The ability to pattern arrays of multicolor light-emitting nanocrystals opens avenues toward the development of optoelectronic devices, including optical displays.

## Teaser

Arrays of site-isolated halide perovskite nanocrystals are synthesized and show size-dependent emission and photovoltaic activity.

## INTRODUCTION

Because of the unusual electronic structures and lattice dynamics of halide perovskites, these materials have been explored extensively in the field of optoelectronics (*1*). Several classes of halide perovskites, including organic-inorganic hybrid crystals (*2*), all-inorganic crystals (*3*), and layered crystals (*4*), have been identified as promising materials for fabricating solar cells (*5*), light-emitting diodes (LEDs) (*6*), lasers (*7*), and radiation detectors (*8*). When the crystal size is reduced below the micrometer range, however, the relationship between crystal dimensions and physicochemical properties is not clear and often debated (*9*). This is, in part, due to the difficulty in preparing site-isolated, high-quality nanocrystals that have defined compositions, sizes, and locations, a capability that would enable the establishment of structure-function relationships at the single-particle level. In addition, the challenge of miniaturizing halide perovskites in a site-specific manner hinders the integration of these materials into micro- and nano-optoelectronic devices (*10*). Conventional lithography techniques are largely incompatible with halide perovskites due to the poor chemical stability of these materials in many solvents required for photolithography (*11*). Emerging patterning tools such as lithographically defined seed conversion (*12*, *13*), inkjet printing (*14*), and templated crystal growth (*15*–*17*) have yielded micrometer- and submicrometer-sized structures in polycrystalline and relatively large single-crystalline states, but no method yet exists for synthesizing arrays of high-quality halide perovskite nanocrystals in a multiplexed manner. Such capabilities and arrays, in principle, not only would enable one to fabricate devices that rely on a single particle but also allow one to investigate the relationship between crystal structure, composition, dimensions, and properties.

Here, we describe a novel way of using polymer pen lithography (PPL) (*18*) to synthesize halide perovskite nanocrystal arrays spanning a variety of substrates over square centimeter areas. By systematically studying the photoluminescence (PL) emission from single nanocrystals as a function of size with controlled temperature, atmosphere, excitation power, and excitation energy, we discovered that the band edges of these nanocrystals are defined by lattice imperfections, which yield multiple independent emission modes. The decreased reabsorption of higher-energy photons leads to an overall blue shift in the PL as the nanocrystal size is reduced, despite not reaching the quantum confinement regime. This result suggests that the optoelectronic responses measured from nanocrystals may better reflect their intrinsic material properties, at least with some types of characterization methods, as opposed to those associated with bulk or thin-film materials. Last, the methodology is general and has been expanded to a library of halide perovskites, which differ in both composition and structure, including a layered Ruddlesden-Popper phase. We show that properly designed nanocrystal arrays synthesized by PPL can constitute RGB pixels or function as single-nanocrystal solar cells, potentially paving the way toward the creation of new types of multicolor optical displays and novel light-harvesting devices.

## RESULTS

### Size-controlled synthesis of individual halide perovskite nanocrystals at defined locations

**Fig. 1:**
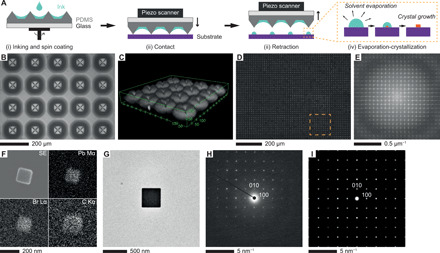
PPL-directed synthesis of MAPbBr_3_ nanocrystal arrays via nanoreactor formation, evaporation, and subsequent crystallization. (**A**) Schematic illustration of the synthesis process for the halide perovskite nanocrystal arrays. (**B**) Optical micrograph of an inked PPL array used for patterning. (**C**) Three-dimensional (3D) confocal microscopy image of PPL pens loaded with dye-labeled inks; note that the pens are not visible because they are not dye-labeled, but the circular reservoirs surrounding each pen are both observable and uniform. Length unit: μm. (**D**) Fluorescence micrograph of a uniform MAPbBr_3_ nanocrystal dot array on a hexamethyldisilazane (HMDS)–modified Si wafer. Dashed box denotes a pattern generated by one polymer pen. (**E**) Fourier transform of (D). (**F**) SEM image and EDS maps of a single nanocrystal. (**G**) TEM image of a nanocrystal. (**H** and **I**) Electron diffraction [experimental (H) and simulated (I)] along the [001] zone axis of the nanocrystal in (G).

In a typical synthesis, an ink solution composed of halide perovskite precursors (e.g., AX and PbX_2_ for the lead halide perovskite APbX_3_, where A is either an organic or inorganic cation and X is a halide anion) dissolved in a mixture of dimethyl sulfoxide (DMSO) and sulfolane was spin coated onto an array of ~1000 polydimethylsiloxane (PDMS) micro-pyramidal pens (pen array) (Fig. 1A, i). Unlike molecular or polymer inks used in conventional PPL, the liquid organic inks accumulate around the base of each pyramid due to the high surface tension and low viscosity of the ink and serve as a reservoir for continuous inking (Fig. 1, B and C). Once the arrays were inked, >100,000 droplet nanoreactors were deposited across a variety of substrates (Fig. 1A, ii and iii). Because of the high surface-to-volume ratio, these nanoreactors readily evaporate within seconds, which leads to the nucleation and growth of individual halide perovskite nanocrystals (iv). As a proof of concept, methylammonium lead bromide (MAPbBr_3_) nanocrystals, which exhibit strong PL (Fig. 1D and fig. S2), were synthesized on silicon substrates. In this case, each of the PDMS pyramidal pens created 121 crystals covering an ~0.024-mm^2^ area (yellow dashed box in Fig. 1D), and the entire substrate is covered with a highly ordered, periodic array as evidenced by the Fourier transform of the fluorescence micrograph (Fig. 1E). The morphology and chemical composition of the crystals were determined using scanning electron microscopy (SEM) and energy-dispersive x-ray spectroscopy (EDS) elemental mapping [note that Pb and Br are uniformly distributed throughout the individual nanocrystals (Fig. 1F)]. Atomic force microscopy (AFM) shows that these nanocrystals have a typical width-to-height ratio of ~3:1 (fig. S3). Transmission electron microscopy (TEM) imaging and selected-area electron diffraction (SAED) of the nanocrystals synthesized on a 15-nm-thick silicon nitride membrane confirm that the nanocrystals are single crystalline (Fig. 1, G and H). The SAED pattern along the [001] zone axis, along which a rectangular projection is observed for the nanocrystal, matches the simulated diffraction pattern for a cubic perovskite structure (Fig. 1I) (*19*). It is critical to note that the ink mixture used in these experiments has a low volatility and remains stable on the pen arrays for at least an hour of continuous patterning. The low volatility is important in generating high-quality single nanocrystals as higher-volatility mixtures did not yield comparable results (fig. S4).

**Fig. 2:**
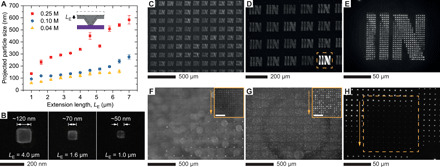
Synthesis of MAPbBr_3_ nanocrystals with controlled particle size, pattern geometry, and substrate choices. (**A**) Size of nanocrystals synthesized from an individual polymer pen as a function of initial ink concentration and extension length on an HMDS-modified Si wafer. Particle sizes were defined as the square root of the projected areas from SEM images. Error bars represent SDs. The extension length, *L*_E_ (defined in the inset), was controlled by an AFM. (**B**) SEM images of nanocrystals synthesized using 0.04 M ink and various extension lengths. The smallest nanocrystals were ~50 nm as determined by SEM. (**C** to **E**) Fluorescence micrographs showing grayscale patterning of the “IIN” logo on an HMDS-modified Si wafer enabled by control of the extension length and subsequent tip flattening (increases feature size). Inset in (D) is the original grayscale pattern design. (**F** to **H**) Fluorescence micrographs of large-scale size-gradient patterns on various substrates: ITO-coated glass (F), a glass slide (G), and silicon nitride thin film [(H) the dashed box outlines the freestanding silicon nitride; thickness = 15 nm]. Arrows indicate the direction of decreasing *L*_E_ for each polymer pen. Insets in (F) and (G): magnified image of an array generated by one polymer pen; scale bars: 50 μm.

In addition to controlling the location of individual nanocrystals, this method enables one to tune the crystal size by controlling both the initial precursor concentration in the ink and the extension length of the PDMS pens against the substrate (Fig. 2A and figs. S5 and S6). Using an initial ink concentration of 0.04 M and extending the PDMS pens only 1 μm result in the formation of ~50-nm MAPbBr_3_ nanocrystals, as evidenced by SEM (Fig. 2B). In principle, the synthesis of even smaller site-isolated nanocrystals should be possible; however, such structures are difficult to characterize and analyze using microscopy techniques. Because the PL intensity scales as a function of nanocrystal size, one can use this size tunability to create grayscale images at the microscale (Fig. 2, C to E). In addition, because this technique is substrate versatile, it can be used to generate comparable patterns on conductive indium tin oxide (ITO)–coated glass, glass slides, and silicon nitride membranes (Fig. 2, F to H, and figs. S7 and S8). However, the single nanocrystal per site yield and corresponding crystal quality depend on the roughness of the substrate. For example, for ITO with a root mean square (RMS) roughness (*R*_g_) of 2.97 nm, multiple particles in each nanoreactor were typically observed, whereas ITO substrates with an *R*_g_ of 0.62 nm had a single-particle yield close to 100% (figs. S8 and S9). In addition, while large-area patterning with a controlled size gradient is possible on glass slides, as evidenced by dark-field (DF) scattering measurements (fig. S7E), the PL of these particles is nonuniform, suggesting poor crystal quality (Fig. 2G).

### Size dependence of single-nanocrystal PL emission

**Fig. 3:**
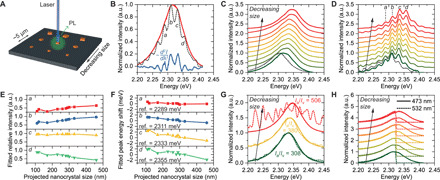
Size-dependent PL from individual MAPbBr_3_ nanocrystals. (**A**) Schematic of PL collection from a single nanocrystal in a location-encoded, size-gradient nanocrystal array. (**B**) HRPL spectrum of an ~460-nm nanocrystal (projected size determined by SEM, red solid curve). Multiple sub-peaks are revealed by both the second derivative of intensity (*I*) over wavelength (λ) (blue solid curve) and Richardson-Lucy deconvolution (dashed curve). a.u.: arbitrary units. (**C** and **D**) HRPL spectra (C) and deconvolution results (D) for a series of nanocrystals of decreasing size (from ~460 to ~110 nm). The spectrum of a bulk crystal is shown as a gray dashed curve for reference. (**E** and **F**) Quantification of peak intensity (E) and center energy (F) of four sub-peaks (*a*, *b*, *c*, and *d*) identified in (D). (**G**) PL spectra of nanocrystals exposed to air (solid curves) or in vacuo (dashed curves, bandpass-filtered for noise reduction). Peak intensity ratios (air versus vacuum) before normalization are given as *I*_a_/*I*_v_. (**H**) HRPL spectra of nanocrystals excited with a 473-nm (~2.62 eV, solid curves) or a 532-nm (~2.33 eV, dashed curves) laser.

To understand the PL properties of individual MAPbBr_3_ nanocrystals of different sizes, we prepared size-gradient nanocrystal arrays with an interparticle spacing of ~5 μm on silicon wafers. Single-nanocrystal emission spectra were collected by focusing the excitation laser onto an ~2-μm spot around each nanocrystal (Fig. 3A) and then correlated with nanocrystal size, as determined by SEM. Unexpectedly, high-resolution PL (HRPL) spectroscopy reveals an emission peak that contains multiple shoulders, as evidenced by the peaks in the second derivative of the spectra (Fig. 3B), which do not originate from the spectrometer (fig. S10). This observation indicates that multiple emission modes may be present. Because no prior knowledge of these modes is available, a direct fit of the HRPL spectra using multiple peak functions is unreliable. As such, we assumed that each mode could be described by an arbitrary peak broadened by a Gaussian point spread function, whereby each spectrum was iteratively deconvolved using the Richardson-Lucy algorithm (Fig. 3B, dashed curve; see also text S1) (*20*, *21*). The shoulders in the HRPL spectrum can be decomposed into multiple emission modes, and their relative peak intensities (when normalized) still match with the envelope shape of the overall HRPL spectrum when the intensity is adequately higher than the noise level. The presence of multiple modes suggests that the band edges in these materials are defined by various lattice imperfections, such as emissive defects (*22*, *23*) and lattice distortion (*24*, *25*). Such a highly defective halide perovskite lattice may no longer be considered continuous; it may not have the well-defined band edges typically observed with large single crystals.

Using this high-throughput approach, we studied the emission from nanocrystals as a function of size from ~460 to ~110 nm. Smaller nanocrystals show an HRPL spectrum blue-shifted to higher energies (Fig. 3C), even though the dimensions of all these nanocrystals are well above the Bohr radius in MAPbBr_3_ that is required for quantum confinement (*26*). Similar blue shifts have been observed in polycrystalline thin films and microstructures of halide perovskites; however, their origin is under debate with several proposed explanations. These pertain to surface depletion (*27*), surface emission (*28*), substrate-induced strain (*29*–*32*), free carrier formation (*33*), and photon reabsorption (*34*). To gain further insight into this size-dependent emission phenomenon, we deconvolved spectra from nanocrystals of different sizes to study the peak energies and relative intensities of all modes (Fig. 3D). The peak positions for all modes are almost the same; however, their relative intensities vary, resulting in the apparent blue shift of the overall emission. Quantitatively, four major modes labeled as *a*, *b*, *c*, and *d* were selected for comparison. When all deconvolved spectra were normalized to the range [0, 1], the relative intensity of modes *a* and *b* decreased as crystal size decreased, while mode *d* increased (Fig. 3E). The peak energies of all four modes exhibit a slight blue shift on the order of a few millielectron volts (Fig. 3F), far below the observed overall PL blue shift for the single nanocrystals of different sizes measured here or for the polycrystalline structures reported in the literature (*27*, *35*). Note that all these modes are closely correlated to the modes present in the bulk crystal, suggesting that they are intrinsic to the crystal and share the same physical origin. These results provide strong evidence that two different types of potentially size-dependent effects exist in halide perovskite nanocrystals: surface depletion–constraint quantum confinement and substrate-induced strain [internal pressure on the megapascal scale (*36*); note that heteroepitaxy is not relevant in this study, as evidenced by the SEM images] are potentially responsible for the slight blue shift of each emission mode. On the other hand, the overall PL shift as a function of crystal size is a result of the systematic intensity modulation of these modes, which has a different physical origin (vide infra).

To reveal the nature of these multiple emission modes that show size-dependent intensity modulation behavior, we systematically analyzed single-nanocrystal emission from crystals with different structures and in different environments. By placing the nanocrystal arrays in high vacuum (<~10^−4^ Pa), deep traps on the surface that are usually blocked by oxygen and water molecules were exposed (*37*), which resulted in a substantial reduction in PL intensity (by a factor of >300; Fig. 3G). The emission peak energy is almost unchanged in the presence or absence of the blocker molecules (in air versus in vacuo), suggesting that the emission modes originate from the interior of the crystals and that surface defects are not involved significantly. Moreover, we partially excited the nanocrystals using an ~2.33-eV laser and compared the HRPL spectra with the fully excited ones to reveal the relationship among different emission modes. The HRPL spectra in the <2.32-eV (filter cutoff) region are unchanged from the fully excited spectra, suggesting that the energy states associated with these emission modes have relatively fixed densities and are independent from one another. We further confirmed that this multimode, size-dependent emission behavior is not a result of organic cation rotation (*38*) or DMSO insertion (*39*) in the MAPbBr_3_ crystals, because all-inorganic CsPbBr_3_ nanocrystals and DMF (*N*,*N*′-dimethylformamide)–derived MAPbBr_3_ polycrystals both show similar effects (figs. S13 and S15). Together, the data are consistent with two plausible pathways: (i) a change in the relative density of emissive states or (ii) a redistribution of the emission intensities at different energies induced by crystal size variation.

**Fig. 4:**
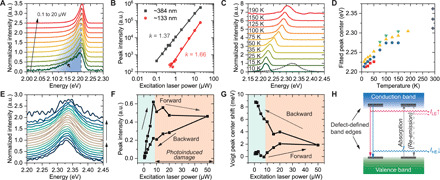
Excitonic properties of single MAPbBr_3_ nanocrystals. (**A**) PL emission of a ~384-nm nanocrystal at 10 K in vacuo excited by a 442-nm laser of varying power. A low-energy tail (indicated by the blue shading and arrow) appears at low excitation power. (**B**) Laser power–dependent PL peak intensity of an ~384-nm (black) and ~133-nm (red) nanocrystal at 10 K. (**C**) Temperature-dependent PL emission of an ~129-nm nanocrystal in vacuo. Its PL spectrum at 292 K in atmosphere is shown as a dashed curve as reference. (**D**) Fitted peak energy values of dominant sub-peaks deconvolved from the spectra in (C). Data points with the same color indicate possibly related sub-peaks at different temperatures. Dominant sub-peak energies from room temperature PL measurements are shown as purple diamonds for reference. (**E**) PL emission of an ~550-nm nanocrystal at room temperature in atmosphere excited by a 442-nm laser of changing power (bottom to top: increasing from 0.5 to 50 μW and then decreasing to 0.5 μW). The spectra were bandpass-filtered for noise reduction. (**F** and **G**) Quantification of peak intensity (F) and center energy shift (versus 2326 meV) fitted by a Voigt function (G) of the spectra in (E). (**H**) Possible excitonic pathways that result in the observed size-dependent emission.

To identify the most probable explanation for the size-dependent emission behavior, we studied the excitonic properties of single MAPbBr_3_ nanocrystals in detail. When cooled to 10 K in vacuo, individual MAPbBr_3_ nanocrystals exhibit well-defined emission depending on the power of the focused excitation laser (Fig. 4A). As the laser power increases from 0.1 to 20 μW, the lower-energy tail in the emission peak indicative of the formation of bound excitons gradually diminishes. The intensity of the main emission peak (*I*) follows a power law against the excitation power (*P*)

$$I\propto P^{k}$$ (1)

with exponent values 1 < *k* < 2 (Fig. 4B), which confirms that the emission is predominantly excitonic (*40*) and rules out nonlinear optical generation in this system. When heated between 100 and 150 K, a gradual transition in emission energy was observed (Fig. 4, C and D), consistent with a phase transition (*41*, *42*). Multi-peak features are present at all temperatures and can be deconvolved below ~200 K when the signal-to-noise ratio is sufficient. These results suggest that the multiple modes are associated with defects intrinsic to the crystal. We further studied the emission from single nanocrystals at room temperature in the atmosphere, excited by cyclically varying excitation power, and a hysteresis loop was observed in the emission intensity (Fig. 4, E and F). Specifically, when the laser power is higher than ~10 μW, PL intensity from the nanocrystal decreases due to photoinduced damage. The peak energy shows almost no change before the laser power reaches the damage threshold when the process is dominated by free exciton-like emission with a power-law slope *k* = 1.14 (fig. S17). We did not observe evidence of an additional bound exciton peak at low excitation power (Fig. 4E). These results are consistent with the interpretation in micrometer-sized and bulk crystals that various intrinsic defect states exist in proximity to the band edges (*22*, *23*) and that deep traps are protected due to screening (*24*, *25*). In addition, the damage-induced peak shift is also less than 10 meV (Fig. 4G), much smaller than the size-induced blue shift that spans tens of millielectron volts. As the emission modes that constitute the PL peak are independent of each other (Fig. 3H), these results suggest that the hot carrier recombination (band filling) effect (*43*) or defect density variation are not the main contributors to the size-dependent emission in halide perovskite nanocrystals. As a result, a redistribution of emission intensity due to the interaction between emitted photons and the crystal, i.e., photon reabsorption and possible photon recycling (*44*), is most likely responsible for this size-dependent behavior. The overall PL energy shift of ~26 meV in the nanocrystals (crystal size from ~460 to ~110 nm, estimated thickness from ~150 to ~40 nm; Fig. 3C) is consistent with the depth-dependent cathodoluminescence energy shift due to reabsorption, as reported in the literature (*45*).

Together, we conclude that two factors primarily cause the size-dependent energy shift of the PL peaks for halide perovskite nanocrystals: (i) defects at the noncontinuous electronic band edges result in excitonic emissions with varying energies, and (ii) the reabsorption of higher-energy photons changes the intensity distribution of these emission modes (Fig. 4H). For larger crystals, photons emitted by higher-energy modes are more efficiently reabsorbed, creating electron-hole pairs that typically relax to lower-energy states nonemissively and causing a decrease in the intensity of higher-energy modes (*I*_HE_). In addition, the relative intensity of the lower-energy modes (*I*_LE_) in larger crystals might be exaggerated further due to re-emission (*44*). It is important to note that the wavelength of the emitted photons (typically 520 to 550 nm) is larger than the nanocrystal dimensions involved in this study (typically 100 to 550 nm). Therefore, the photon energy transfer in nanocrystals is highly localized unlike what occurs in the bulk and microcrystals, which is typically described by a semiclassical light propagation model (*35*). Critically, this dominant pathway is independent of the surface or strain effects, which cause a minimal energy shift (on the order of 10^0^ meV) as a function of crystal size.

### Nanocrystal libraries and devices of halide perovskites

**Fig. 5:**
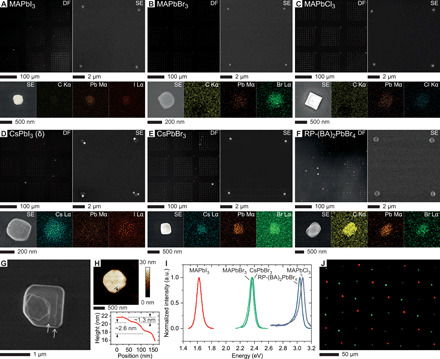
Multicolor halide perovskite nanocrystal libraries. (**A** to **F**) Arrays of halide perovskite nanocrystals synthesized on fluoropolymer-modified ITO-coated glass characterized by optical DF microscopy, SEM, and EDS elemental mapping: MAPbI_3_ (A), MAPbBr_3_ (B), MAPbCl_3_ (C), nonemissive δ-CsPbI_3_ (D), CsPbBr_3_ (E), and layered Ruddlesden-Popper butylammonium lead bromide [RP-(BA)_2_PbBr_4_] (F). (**G**) SEM image of a layered RP-(BA)_2_PbBr_4_ nanocrystal with multiple steps on the surface (arrows). (**H**) AFM height image of a layered RP-(BA)_2_PbBr_4_ nanocrystal (top) and step profile of the region indicated by the black lines (bottom). Average height values along the parallel lines are given to minimize sampling inconsistency. (**I**) Representative PL spectra of individual nanocrystals of different compositions. The spectrum for MAPbCl_3_ was bandpass-filtered to reduce the noise. (**J**) Merged-channel confocal fluorescence image of tricolor nanocrystal pixel arrays composed of MAPbI_3_ (red), MAPbBr_3_ (green), and MAPb(Br_0.4_Cl_0.6_)_3_ (blue).

By changing the chemical precursors, AX and PbX_2_ (different cations, A, and halides, X), a library of halide perovskite nanocrystal arrays can be synthesized and studied. Specifically, nanocrystals of solution-processable halide perovskites MAPbI_3_, MAPbBr_3_, MAPbCl_3_, CsPbI_3_, and CsPbBr_3_ were synthesized using PPL and the appropriate precursor inks (Fig. 5, A to E). In addition to these “3D” (three-dimensional) halide perovskites, a layered Ruddlesden-Popper halide perovskite was synthesized in nanocrystal array format with butylammonium bromide (BABr) and PbBr_2_ as the precursors in the inks (Fig. 5F and fig. S22). SEM reveals the presence of thin steps on the surface of the rectangular nanocrystals, indicative of the targeted 2D layered structure. Critically, AFM identifies single- and double-layer step heights of ~1.3 and ~2.6 nm, consistent with the reported layer thickness in bulk crystals (1.4 nm) and single-layer sheets (1.6 nm) (*46*). With the exception of nonemissive δ-CsPbI_3_ that is formed due to thermodynamic limitations at room temperature, all halide perovskite nanocrystals exhibit well-defined PL emission (Fig. 5I). By sequentially patterning MAPbI_3_ (red), MAPbBr_3_ (green), and MAPb(Br_0.4_Cl_0.6_)_3_ (blue) nanocrystals on the same substrate, light-emissive RGB pixel arrays with all three colors were synthesized (Fig. 5J and fig. S23). This capability for synthesizing position-defined halide perovskite nanocrystals with controlled emission wavelengths points toward a new way of creating multicolor micropixels, potentially suitable for high-density display technologies.

**Fig. 6:**
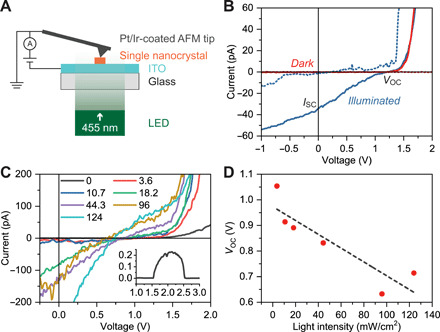
Single-nanocrystal photovoltaics in an ambient environment. (**A**) Schematic of a hole-transporter-free single-nanocrystal solar cell using conductive AFM. (**B**) Current-voltage curves for a MAPbBr_3_ nanocrystal in the dark (red) or illumined by a 455-nm LED light of ~3.6 mW/cm^2^ (blue). *V*_OC_: open-circuit voltage; *I*_SC_: short-circuit current. Forward (dotted) and backward (solid) scans show significant hysteresis, presumably due to ion migration in the crystal or conditioning of the contact between the probe and crystal. (**C**) Light intensity–dependent photovoltaic response of a MAPbBr_3_ nanocrystal (backward scans). Light intensity unit: mW/cm^2^. Current variation between measurements is mainly attributed to the unstable point contact between the AFM probe and the crystal. LED wavelength: 455 nm. Inset: AFM height profile of the nanocrystal; length unit: μm. (**D**) Open-circuit voltage as a function of light intensity derived from (C) (red dots) and its linear fit (dashed line).

Last, halide perovskite nanocrystals synthesized by PPL can also be used to prepare photovoltaic devices, including miniaturized solar cells. As a proof of concept, a hole-transporter-free (*47*) solar cell was constructed by first patterning a single MAPbBr_3_ nanocrystal on an ITO-coated glass substrate and then connecting its top surface using a Pt/Ir-coated conductive AFM probe (Fig. 6A). An in situ AFM stage was used to illuminate the nanocrystals (455- or 530-nm LED light source) during the experiment (fig. S24). In the dark, no appreciable photocurrent was observed, while illumination under 455-nm LED light (~3.6 mW/cm^2^) immediately triggered measurable photocurrents across the nanocrystal (Fig. 6B), although significant hysteresis was observed between the forward and backward scans, presumably due to ion migration or conditioning of the probe-crystal contact (*48*). This photovoltaic response was observed in all four site-isolated nanocrystals studied with an open-circuit voltage (*V*_OC_) between 1.06 and 1.21 V (fig. S25 and table S1). Under prolonged light illumination, the nanocrystals showed varying responses due to the unstable contact between the AFM probe and nanocrystal, and significant material degradation was observed as the light intensity was increased (Fig. 6C). Specifically, *V*_OC_ drops to <0.8 V when the light intensity exceeds 120 mW/cm^2^, indicating an increase in defect density and highlighting the importance of stabilizing halide perovskite nanocrystals for photovoltaic applications. Similar behavior but with less activity was observed with a 530-nm LED source (fig. S26).

## DISCUSSION

This work is important because it describes a novel, straightforward way of synthesizing and studying perovskite nanocrystals in single crystal or crystal array formats. The utilization of the cantilever-free scanning probe method, combined with the identification of appropriate inks (both perovskite precursors and low-volatility solvents with appropriate viscosity), yields one of the more versatile ways of studying such structures as single compositions/sizes or in combinatorial format, where composition and size (≥50 nm) are library parameters. These unique capabilities have allowed us to systematically investigate the size-dependent emission properties of individual halide perovskite nanocrystals. Such studies have revealed that the defect-defined band edges in single nanocrystals lead to multiple excitonic PL emission modes and that the less efficient reabsorption of higher-energy photons leads to an overall blue shift in the PL energy, despite the fact that the crystal size exceeds the quantum confinement range. This discovery highlights the role of intrinsic defects in nanoscale halide perovskite crystals and that size and defect engineering will become critical in tuning the optoelectronic properties of individual nanocrystals. The properties of nanocrystals, and particularly ones with dimensions larger than tens of nanometers that avoid strong quantum confinement, may better reflect the intrinsic optoelectronic properties of halide perovskites than bulk crystals or thin films due to suppressed photon reabsorption and recycling, at least when certain characterization methods are used. We have further shown that this nanoreactor-based synthetic approach is generally applicable for a wide variety of halide perovskites and can be used to prepare multicolor light-emitting nanocrystal arrays and functional single-nanocrystal solar cells. It is worth noting that more detailed emission properties of halide perovskite nanomaterials may be extracted from these location-encoded nanocrystal libraries using other forms of single-particle spectroscopy techniques and that the device stability can be further optimized through engineering, perhaps through encapsulation with protective layers. Therefore, taken together, the methods reported here not only provide a powerful new platform for elucidating the size-dependent photophysical properties of such materials but also open the door to fabricating functional optoelectronic single-nanocrystal devices for power generation and light emission in integrated micro- and nanoelectronic devices, including robotic and wearable electronic systems.

## MATERIALS AND METHODS

### Synthesis of halide perovskite nanocrystal arrays

In a typical synthesis of halide perovskite ABX_3_ [e.g., A = methylammonium (MA), B = Pb, and X = Br], DMSO and sulfolane in a volume ratio of 7:3 were mixed to form a homogeneous solution. This solvent was used for all syntheses unless otherwise noted. Equimolar amounts of AX (e.g., MABr) and BX_2_ (e.g., PbBr_2_) powders were dissolved in the solution to achieve the target concentration (e.g., 0.04, 0.10, or 0.25 M in terms of ABX_3_) and stirred overnight to form an ink. For the layered halide perovskite, butylammonium lead bromide [(BA)_2_PbBr_4_], BABr, and PbBr_2_ powders were mixed in a 2:1 molar ratio.

Flat substrates, such as silicon wafers, glass, ITO-coated glass, or silicon nitride films, were modified by overnight enclosure in a chamber with vials containing a hexamethyldisilazane (HMDS)/hexane mixture (volume ratio = 1:1). For the synthesis of the halide perovskite nanocrystal libraries, ITO-coated glass was modified by depositing fluoropolymers from CHF_3_ in a reactive ion etching process (figs. S18 and S19) (*49*). This surface treatment prevents the spreading of solvent droplets, which is important for the formation of individual crystals in each nanoreactor.

PDMS pen arrays were fabricated following a published protocol (*50*). The pen array was loaded onto the piezo scanner of an AFM (NX Series, Park System Inc.) or a desktop nanopatterning instrument (TERA-fab M series, TERA-print LLC) and leveled to be parallel to the substrate. The pen array was removed from the instrument, treated by oxygen plasma, and then spin coated with the ink at a spin speed of 2000 to 3000 rpm for 1 min (depending on the pen array size). The pen array was then returned to the instrument, brought in contact with the substrate, and extended a certain length (extension length, *L*_E_). Nanoreactors of the ink were formed on the substrate after retraction of the pen array and allowed to evaporate under atmospheric conditions to form individual halide perovskite nanocrystals.

Bulk crystals used for reference spectra were prepared by drop-casting the inks onto the same substrates. The inks were allowed to evaporate under atmospheric conditions resulting in large crystals.

### Microscopy

The inked pen arrays were imaged using an optical microscope (Zeiss Axio Imager M2) under both the DF and bright-field (BF) conditions with a halogen light source. To visualize the ink distribution on the pen arrays in 3D, inks labeled with Nile blue (10 μM) were spin coated on the pen arrays and imaged using confocal fluorescence microscopy on a Zeiss LSM 800 [objective: 10×/0.30 air; pinhole size = 1 Airy Unit (AU)].

Nanocrystal arrays were imaged using an optical microscope (Zeiss Axio Imager M2) under both DF and fluorescence microscopy conditions with a fluorescence LED illuminator (X-Cite, Excelitas Technologies) with or without pertinent optical filters. Multicolor nanocrystal arrays were imaged using confocal fluorescence microscopy on a Zeiss LSM 800 (objective: 10×/0.30 air or 20×/0.80 air; pinhole size = 1 to 5 AU). The morphology and elemental distribution of the nanocrystals were characterized by SEM on a Hitachi SU8030 equipped with a cold field emission gun (cFEG) operated at 1 to 15 kV and an EDS silicon drift detector (X-Max^N^, Oxford Instruments). The projected size of the particles was measured from SEM images using an automated algorithm described in a previous report (*51*). TEM and electron diffraction were performed on a JEOL JEM-ARM300F equipped with a cFEG operated at 300 kV and a Gatan OneView complementary metal-oxide semiconductor camera. Simulated electron diffraction was generated using the SingleCrystal package (CrystalMaker Software Ltd.) based on a published crystal structure (*19*). AFM was performed on a Bruker Dimension Icon in tapping mode (probe *k* = 42 N/m).

### Single-nanocrystal PL measurements

HRPL was performed on a modified confocal Raman spectrometer (HORIBA LabRAM HR Evolution) with an excitation laser wavelength of 473 or 532 nm. In vacuo and low-temperature PL measurements were performed on a homebuilt confocal microscope setup equipped with a 100× objective lens with a numerical aperture of 0.60 (Nikon T-PLAN SLWD 100×), an excitation laser operated at 442 nm (Kimmon Koha IK5451R-E He-Cd laser), and the samples were loaded in a cryostat (Advanced Research Systems DE-202 cryostat with optical access). Motorized micrometers (one-axis motorized translation stage) were used for 2D scanning capabilities. PL spectra were captured on spectrometers with homebuilt scanning and collection software (Shamrock SR-750, Shamrock SR-303, and iDus DU420A camera unit; software built in LabVIEW). The He-Cd laser unit was operated at 320 nm for ultraviolet (UV) excitation on a similar setup with UV-compatible mirrors and a UV-fused silica aspheric lense. Time-resolved PL was performed on a PicoQuant FluoTime 300 spectrometer connected to a Zeiss Observer Z1m inverted microscope through optical fibers, and a diode pulse laser (PicoQuant LDH-P-C-440M) operated at 440 nm was the excitation source.

### Single-nanocrystal photovoltaics

A single-nanocrystal solar cell was assembled based on a hole-transporter-free design (*47*). Briefly, MAPbBr_3_ nanocrystals were synthesized on ITO-coated glass modified using HMDS. An in situ light-illuminated AFM was built based on a Bruker Dimension Icon with a customized transparent stage allowing for LED light (455 or 530 nm) to illuminate the sample (fig. S24). Conductive Pt/Ir-coated AFM probes were used in contact mode to close the circuit. The voltage/current curves were measured both with and without illumination. For each curve, the height of the AFM probe was adjusted to compensate for any probable height change.

## Supplementary Material

abc4959_SM.pdf

## SUPPLEMENTARY MATERIALS

Supplementary material for this article is available at http://advances.sciencemag.org/cgi/content/full/6/39/eabc4959/DC1
