# Supplementary material for: Halide perovskite nanocrystal arrays: Multiplexed synthesis and size-dependent emission
Source: Sci Adv. 2020 Sep 23;6(39):eabc4959. doi: 10.1126/sciadv.abc4959 (PMC7531881; doi:10.1126/sciadv.abc4959)
Supplement: abc4959_SM.pdf [file abc4959_SM.pdf]

Science Advances
AAAS

advances.sciencemag.org/cgi/content/full/6/39/eabc4959/DC1

Supplementary Materials for

Halide perovskite nanocrystal arrays: Multiplexed synthesis and size-dependent emission

Jingshan S. Du, Donghoon Shin, Teodor K. Stanev, Chiara Musumeci, Zhuang Xie, Ziyin Huang, Minliang Lai, Lin Sun, Wenjie Zhou, Nathaniel P. Stern, Vinayak P. Dravid*, Chad A. Mirkin*

*Corresponding author. Email: v-dravid@northwestern.edu (V.P.D.); chadnano@northwestern.edu (C.A.M.)

Published 23 September 2020, *Sci. Adv.* **6**, eabc4959 (2020)
DOI: 10.1126/sciadv.abc4959

This PDF file includes:

Text S1. Deconvolution of HRPL spectra
Figs. S1 to S26
Table S1

Supplementary Text

Text S1

Deconvolution of high-resolution photoluminescence (HRPL) spectra

In a typical HRPL spectrum, each data point (CCD pixel) represents 0.052 nm in the wavelength space. To preliminarily identify the possible number of peaks in a HRPL spectrum, we first calculated the second derivative after smoothing the spectra using a 2nd-order 50-point Savitzky-Golay filter (Fig. 3B, blue curve). A negative peak in the second derivative (i.e., a positive peak in the $-\frac{d^2I}{d\lambda^2}$ plot) represents a concave turning point in the slope and indicates a shoulder feature. In this example, peaks *a*, *b*, *c*, and *d* are clearly visible, but the center energy of these peaks cannot be determined from the second derivative due to overlap.

Notably, directly fitting such a HRPL spectrum with several peak functions is unreliable. Since we do not have prior knowledge of the peak shapes, the large number of degrees of freedom results in many possible fits that converge with distinct peak shapes, center energies, and relative intensities. Therefore, we used a deconvolution approach with minimal prior assumptions to show the position of each mode. We assumed that each mode is represented by an arbitrary sharp peak (width $\ll$ HRPL peak width) broadened by a Gaussian point spread function (PSF), which together constitute the overall spectrum that contains multiple shoulders. We constructed a 512-point Gaussian window that has an arbitrarily assigned width characterized by the standard deviation, σ_{GW}, and used the Richardson–Lucy algorithm to deconvolve the spectrum (iterations: 50). The choice of a small σ_{GW} decreases the smallest distance between neighboring detectable modes but also exaggerates noise. In this example, the method is consistent in recognizing the major peaks (*a*, *b*, *c*, and *d*) when σ_{GW} is varied between 30 and 50, in agreement with the second derivative results (Fig. S11A). By fitting the deconvolution results using Gaussian functions, the center energy values for these deconvolved peaks were determined (Fig. S11B).

To study if the energy values identified from deconvolution results reflect the original HRPL spectra, we fit the original spectrum with multiple Voigt functions with fixed peak center energies using the deconvolution results. The overall spectrum shape is well reconstructed (Fig. S11C) with residuals less than 0.005 (on a normalized scale in [0, 1]; Fig. S11D). Therefore, we conclude that the peaks and their center energies identified by the deconvolution method can reflect the multiple modes that exist in the original HRPL spectrum.

Supplementary Figures

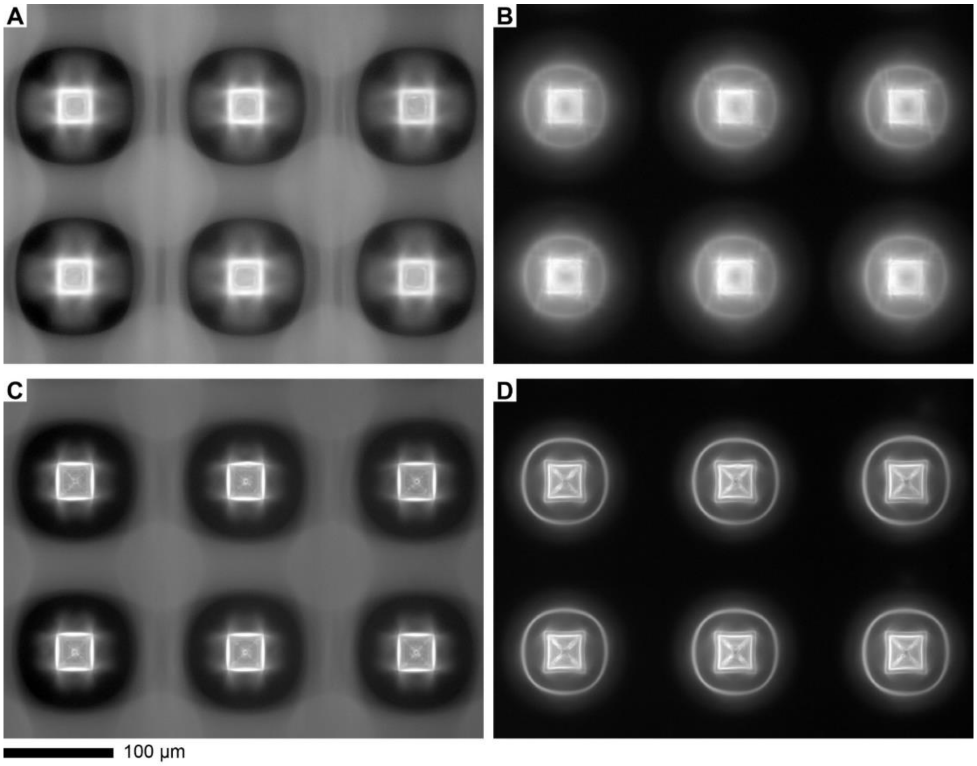

Fig. S1. Optical micrographs of inked PPL tip arrays focused on the base plane (A and B) and tip (C and D). (A) and (C) are bright-field images; (B) and (D) are dark-field images.

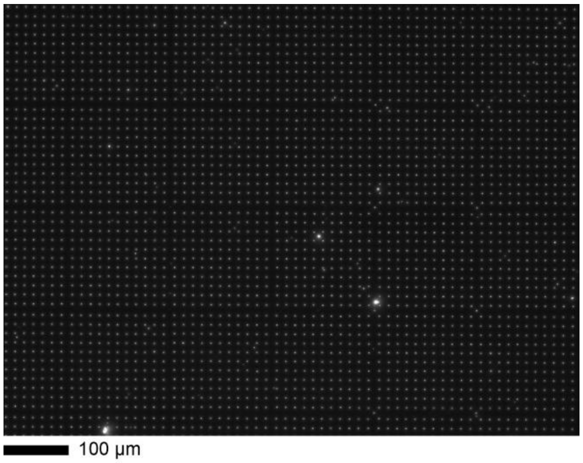

Fig. S2. Dark-field micrograph of a uniform $MAPbBr_3$ nanocrystal dot array (shown in Fig. 1D) on HMDS-modified Si wafer.

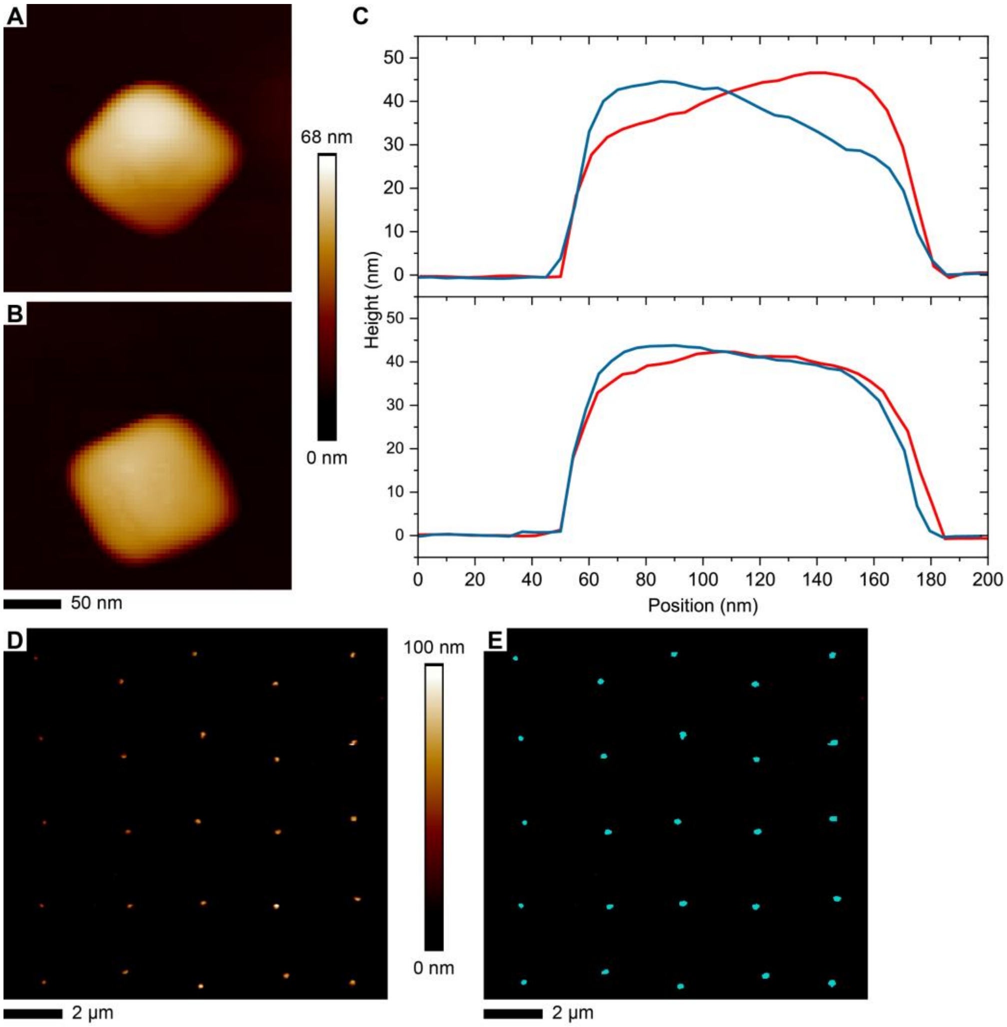

Fig. S3. Topography of $MAPbBr_3$ nanocrystals characterized by AFM. (A and B) AFM height images of two nanocrystals. (C) Cross-sectional height profiles of the two nanocrystals [upper panel is the nanocrystal in (A); lower panel is the nanocrystal in (B)] in two orthogonal directions (red and blue). AFM scans were tilt-corrected. (D) Large-area AFM image and (E) threshold-filtered image for particle statistics (filtered pixels shown in cyan). For the 25 features in this image, mean (± standard deviation) height is 78.6 ± 21.7 nm, and mean diameter is 205.7 ± 23.0 nm. Note that due to the varying particle orientations and limited resolution in large-area AFM scans, these numbers are a rough reflection of the particle size distribution.

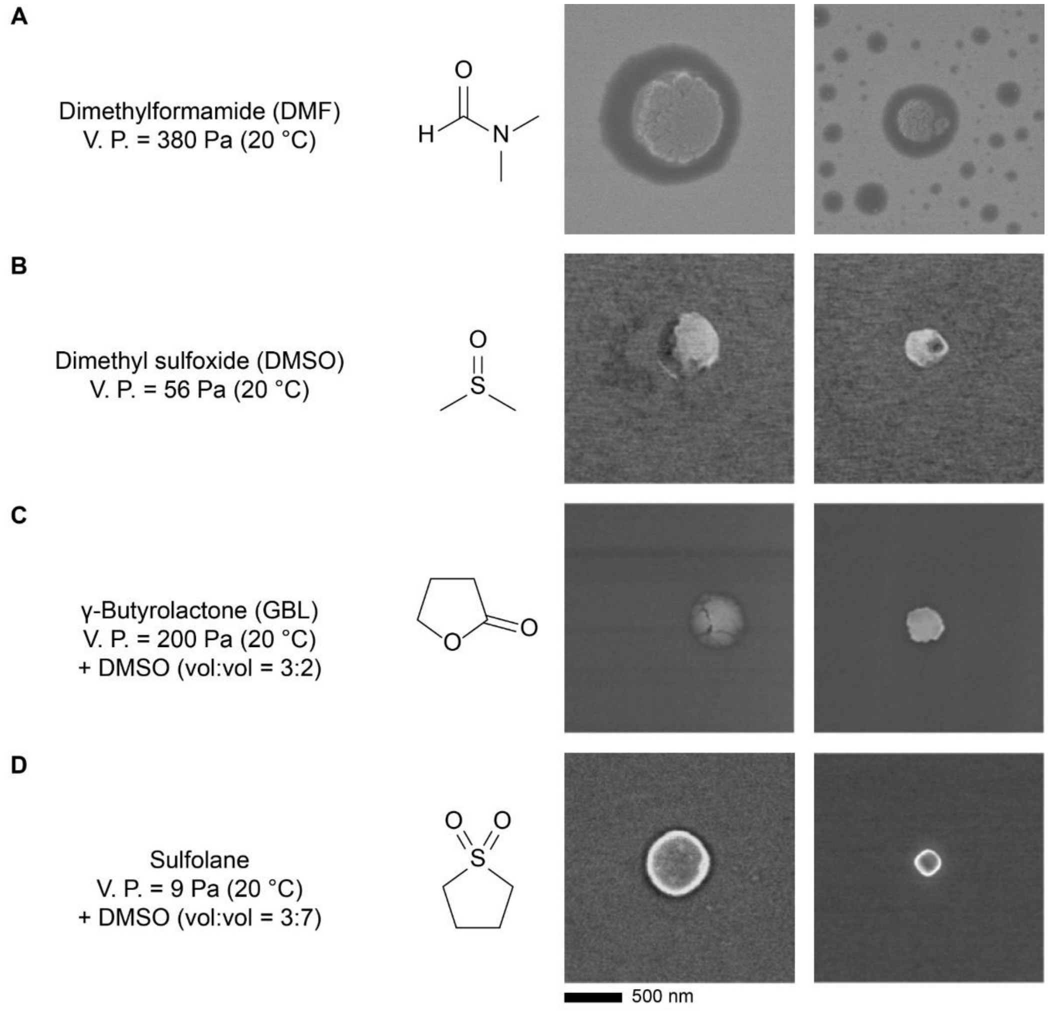

Fig. S4. Effect of solvent on the crystallization of $MAPbBr_3$. Four solvents with different vapor pressure (V. P.) were used to prepare the ink: DMF (A), DMSO (B), a mixture of GBL and DMSO (C), and a mixture of sulfolane/DMSO (D). Right columns show representative SEM images of the crystallization products. All images share the same scale bar of 500 nm.

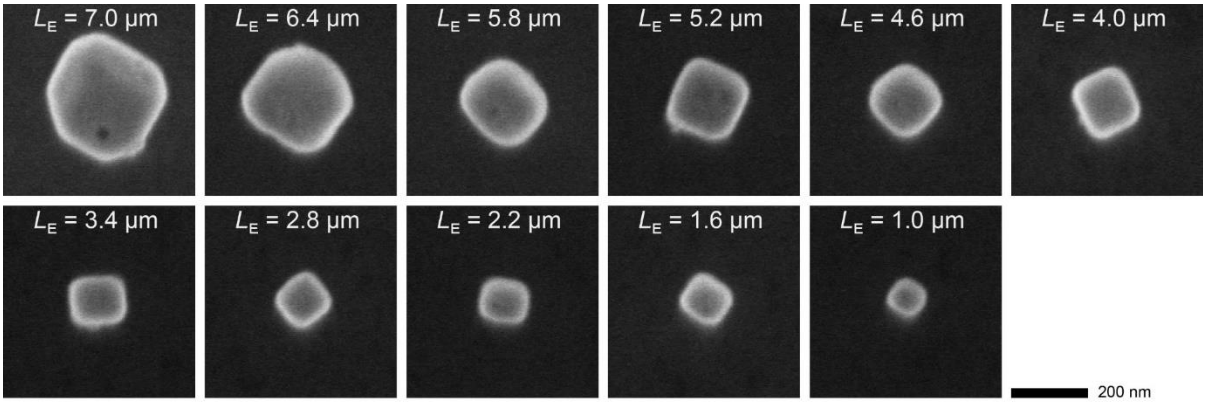

Fig. S5. SEM images of $MAPbBr_3$ nanocrystals where the size of the nanocrystals was controlled by tuning the extension length, L_E, during nanoreactor deposition. Initial ink concentration: 0.1 M in sulfolane/DMSO (volume ratio 3:7). All images share the same scale bar of 200 nm.

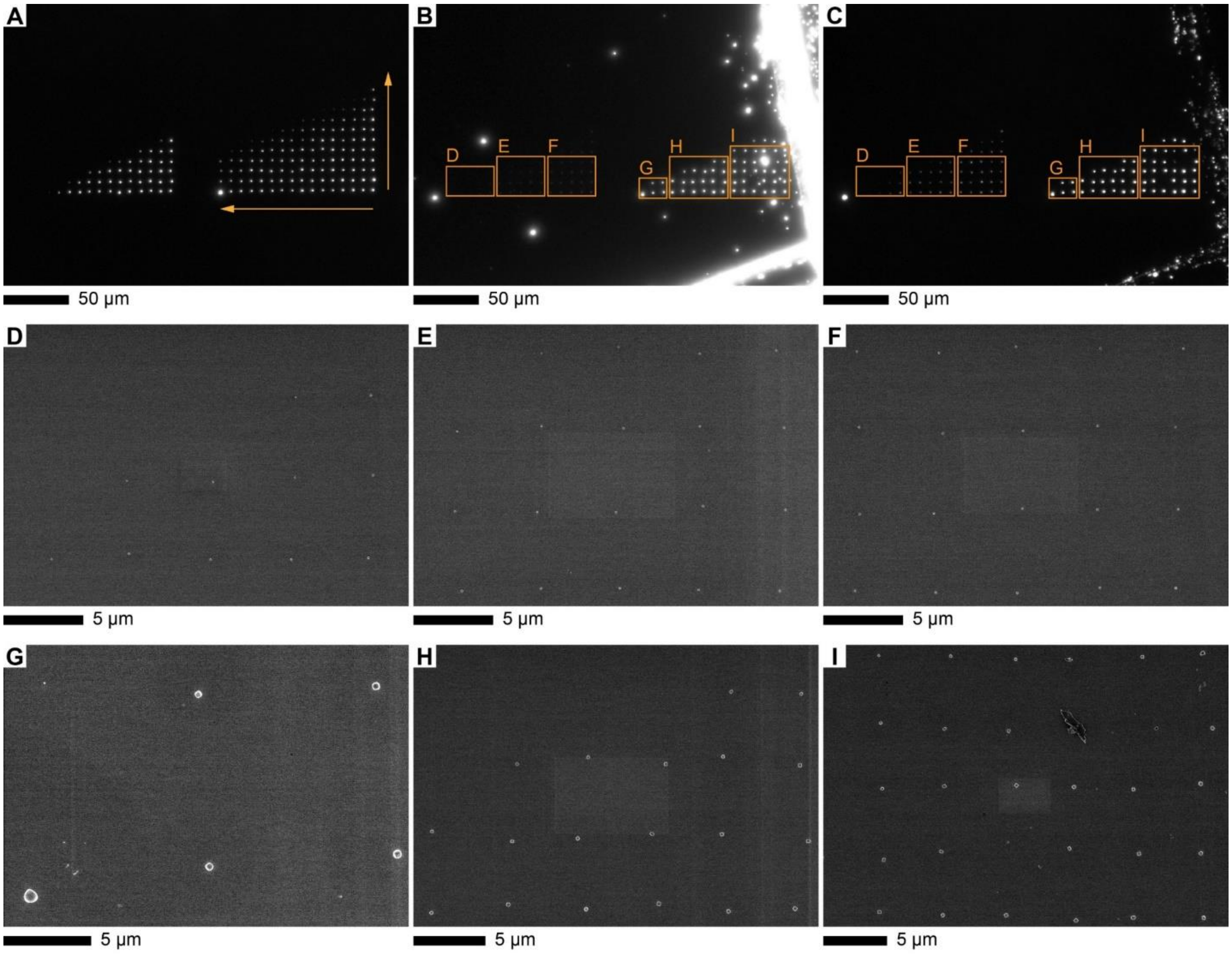

Fig. S6. Double size-gradient $MAPbBr_3$ nanocrystal arrays. (A) Fluorescence micrograph with arrows pointing in the direction of decreasing extension length, L_E (lowest in upper left corner and highest in lower right corner). The difference in the number of features between the patterns from two adjacent polymer pens is due to the intrinsic height variation between these pens (on the order of 1 μm). (B) Dark-field and (C) fluorescence micrograph of two arrays close to scratches on the substrate (bright features on the right). The pattern adjacent to the scratch has significantly larger particle sizes likely due to the damaged substrate and debris, but it still shows a high single-nanocrystal yield. (D to I) SEM images of the nanocrystals in the areas labeled in (B) and (C). Rectangles of bright contrast in the middle of each image are due to electron beam-induced carbon deposition that occurred during focusing.

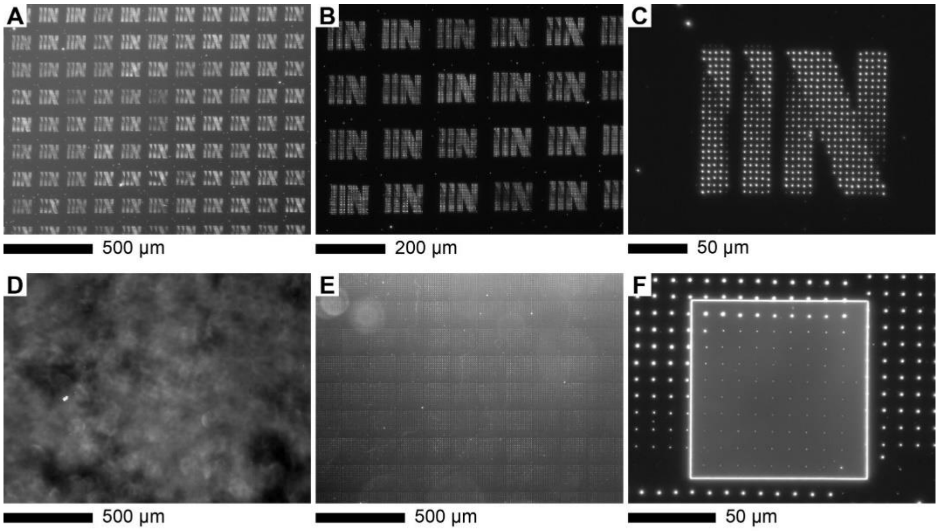

Fig. S7. Dark-field micrographs showing grayscale patterning of $MAPbBr_3$ nanocrystals on various HMDS-modified substrates: (A to C) Si wafer, (D) ITO-coated glass, (E) glass slide, and (F) silicon nitride thin film. The bright square in (F) is the window edge of the freestanding silicon nitride. These images correspond to the fluorescence micrographs in Fig. 2C to H.

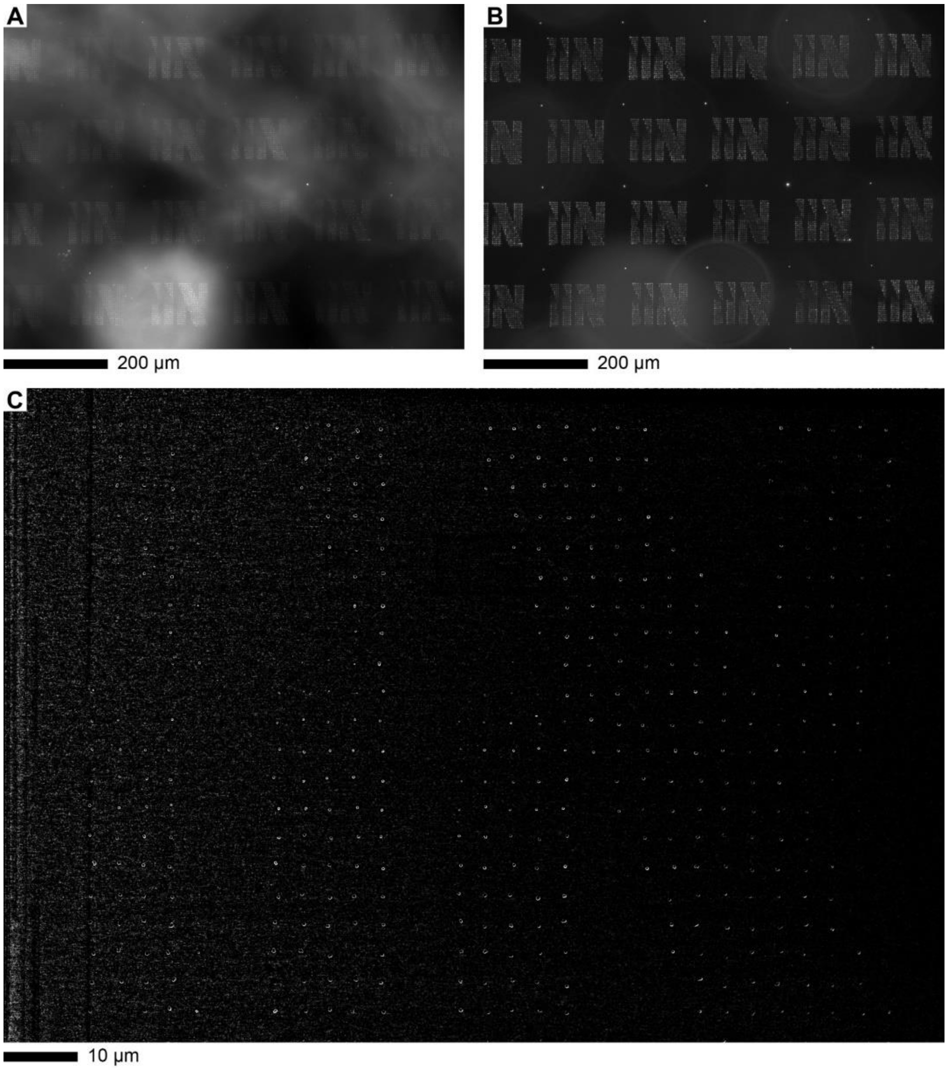

Fig. S8. Grayscale patterning of the "IIN" logo with $MAPbBr_3$ nanocrystals on HMDS-modified ITO-coated glass. **(**A) Dark-field micrograph. (B) Fluorescence micrograph. (C) SEM image.

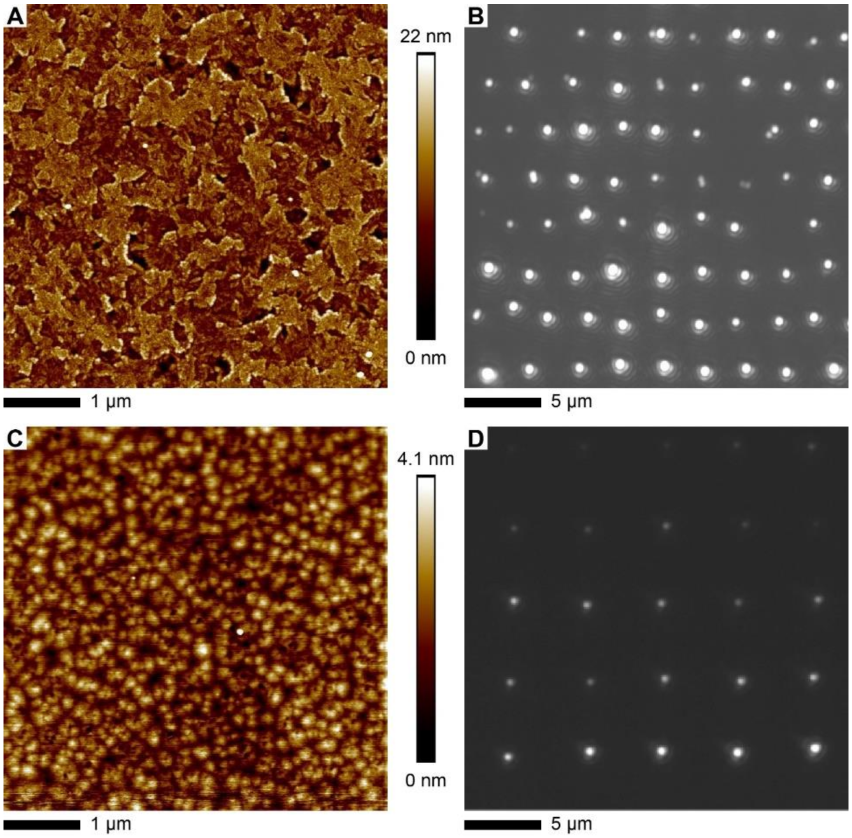

Fig. S9. Effect of substrate roughness on patterning. (A and C) AFM height images for two types of HMDS-modified ITO-coated glass. Surface roughness, R_q, was determined from scans collected under identical conditions (512 × 512 pixels over a 5 × 5 μm area): R_q = 2.97 nm (A) and 0.62 nm (C). (B and D) Optical micrographs showing the patterned nanoparticle arrays on both substrates. Patterns on the rougher substrate (B) exhibit more irregularity and result in the formation of multiple particles per nanoreactor, while the size-gradient patterns on the smoother substrate (D) are more uniform without the formation of multiple particles per nanoreactor.

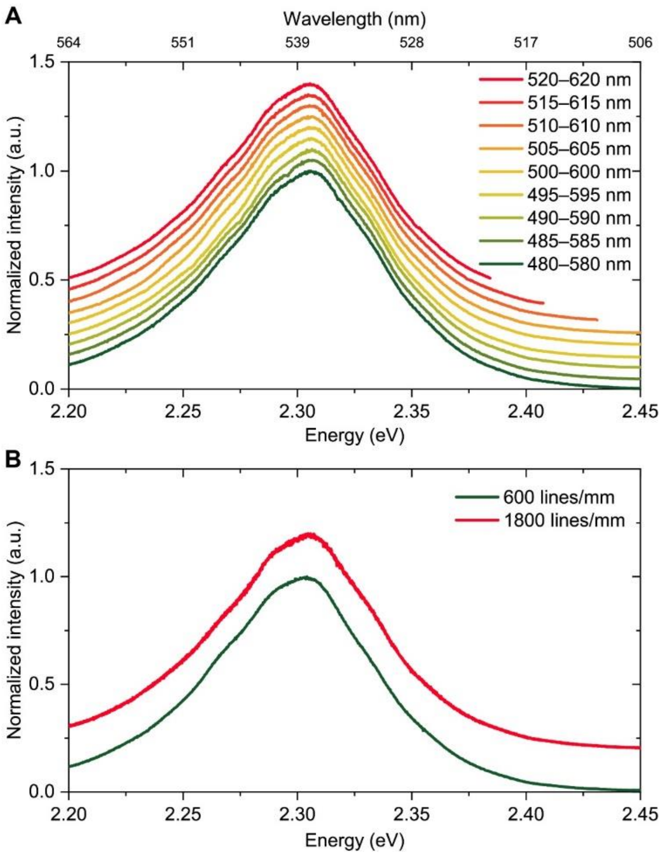

Fig. S10. HRPL spectra collected under different spectrometer setups. (A) HRPL spectra of a $MAPbBr_3$ crystal collect over different detection ranges on the CCD camera. (B) HRPL spectra of a $MAPbBr_3$ crystal collected with two different grating densities. No significant change in the shape of the spectra was observed in either case, suggesting that the multiple sub-peak features in the HRPL spectra are not a result of CCD gain unevenness or an artifact from the diffraction grating.

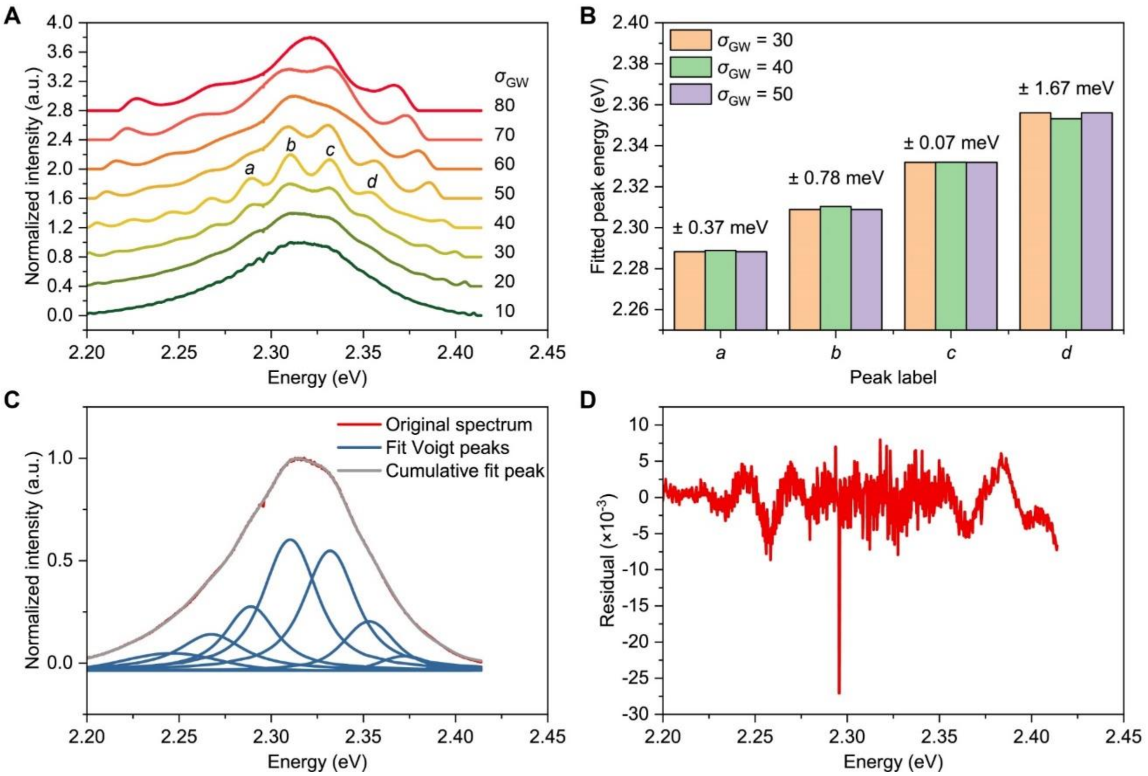

Fig. S11. Robustness analysis of the deconvolution of HRPL spectra. (A) Deconvolution results for the same HRPL spectrum ($MAPbBr_3$, size ~ 460 nm) using a Gaussian window PSF of different width (characterized by the standard deviation σ_{GW}). 50 iterations were run in all cases. (B) Fitted peak energy values of the four peaks (*a*, *b*, *c*, and *d*) in (A). (C) Reconstruction of the original spectrum using Voigt functions with fixed center energy values from the deconvolution results with σ_{GW} = 40. (D) Residual of the fit shown in (C).

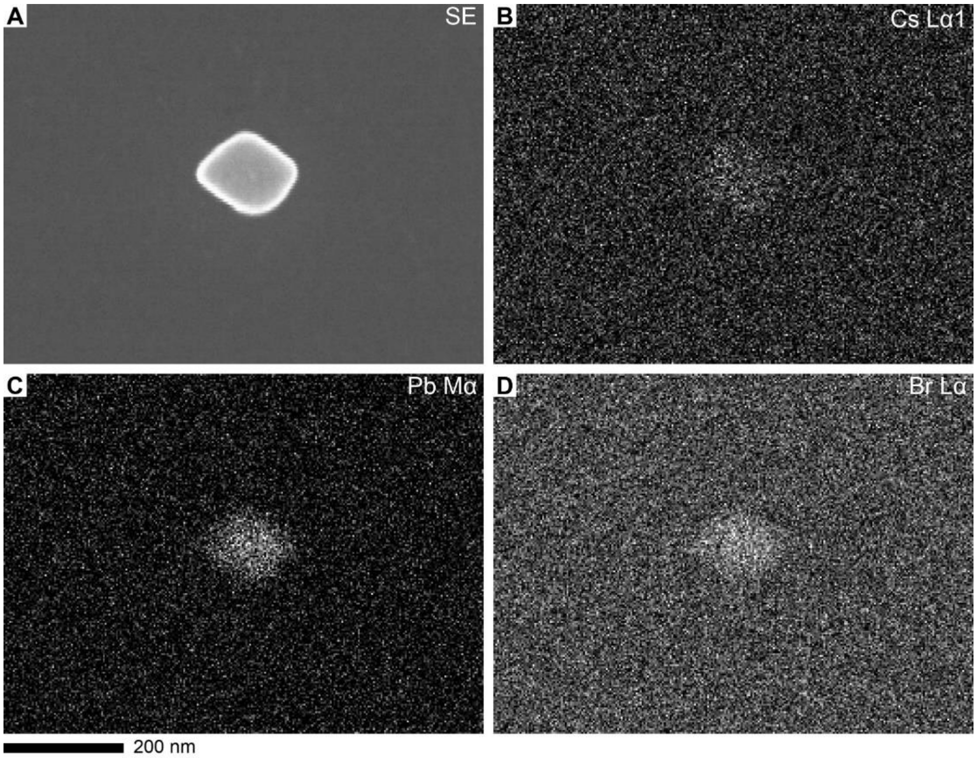

Fig. S12. SEM image and EDS mapping results for a $CsPbBr_3$ nanocrystal. (A) SEM image. (B to D) EDS maps. The substrate is HMDS-modified ITO-coated glass, and the imaging acceleration voltage is 15 kV.

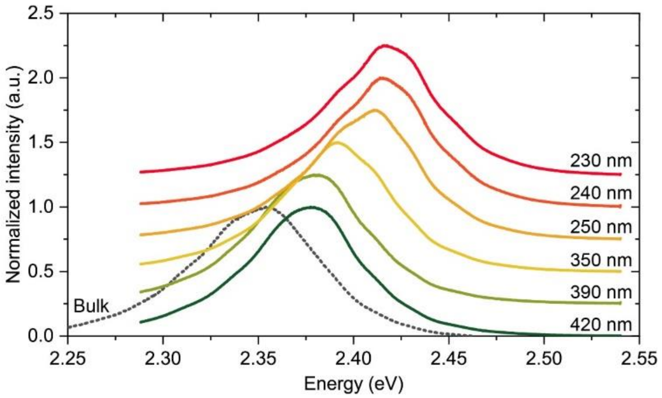

Fig. S13. Size-dependent PL spectra of individual $CsPbBr_3$ nanocrystals of different sizes (measured from SEM images; size accuracy ± ~10 nm). The PL spectrum from a bulk crystal is shown as a reference.

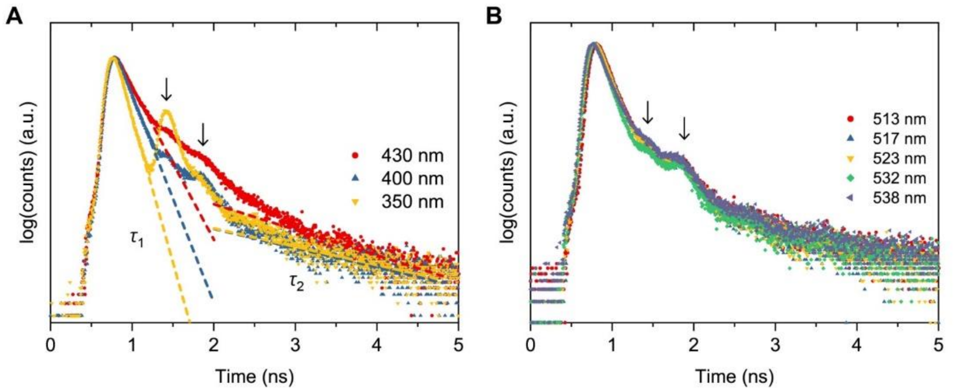

Fig. S14. Transmissive TRPL of $CsPbBr_3$ nanocrystals. (A) TRPL spectra of $CsPbBr_3$ nanocrystals of different sizes detected at 523 nm. Two decay rates (τ_1 and τ_2) are identified by linear fits. (B) TRPL spectra of an individual ~400 nm $CsPbBr_3$ nanocrystal at different detection wavelengths. Arrows in both panels indicate artifact peaks due to internal reflection in the optical path. All spectra were obtained consecutively from the same batch of measurements under the same conditions.

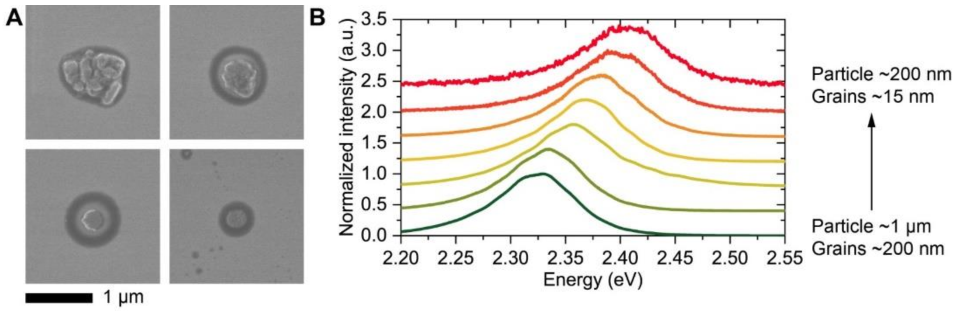

Fig. S15. Emission of polycrystalline $MAPbBr_3$ nanoparticles synthesized using DMF as the solvent. (A) SEM images of polycrystalline $MAPbBr_3$ nanoparticles with decreasing particle and grain size. (B) PL spectra of polycrystalline $MAPbBr_3$ as a function of particle and grain size.

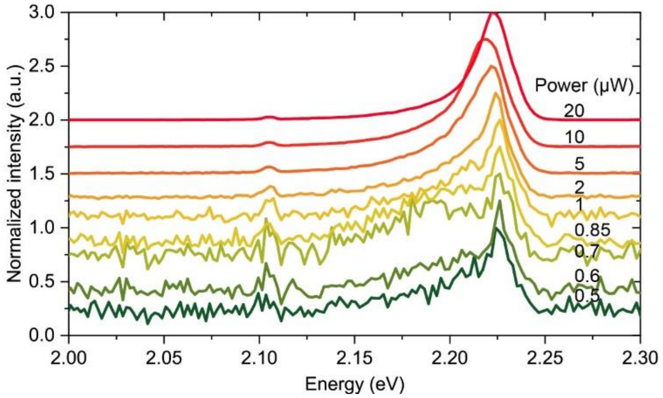

Fig. S16. PL emission of a single ~133 nm $MAPbBr_3$ nanocrystal at 10 K in vacuum excited by a 442-nm laser with varying excitation power.

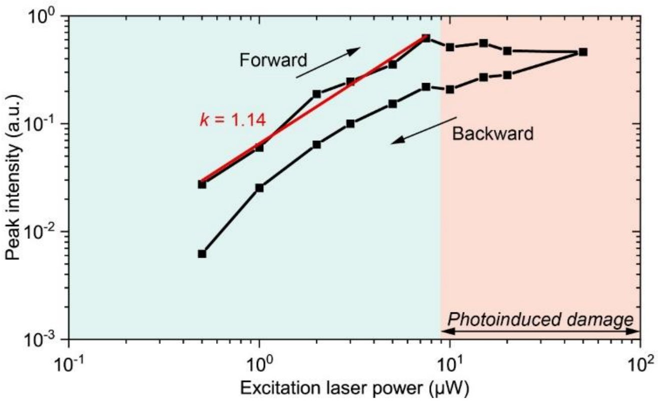

Fig. S17. Log-log scale plot of the PL emission peak intensity of a $MAPbBr_3$ nanocrystal as a function of excitation laser power. The slope of the forward scan before photoinduced damage occurred is $k = 1.14$. This figure is a replot of Fig. 4F in the log-log scale.

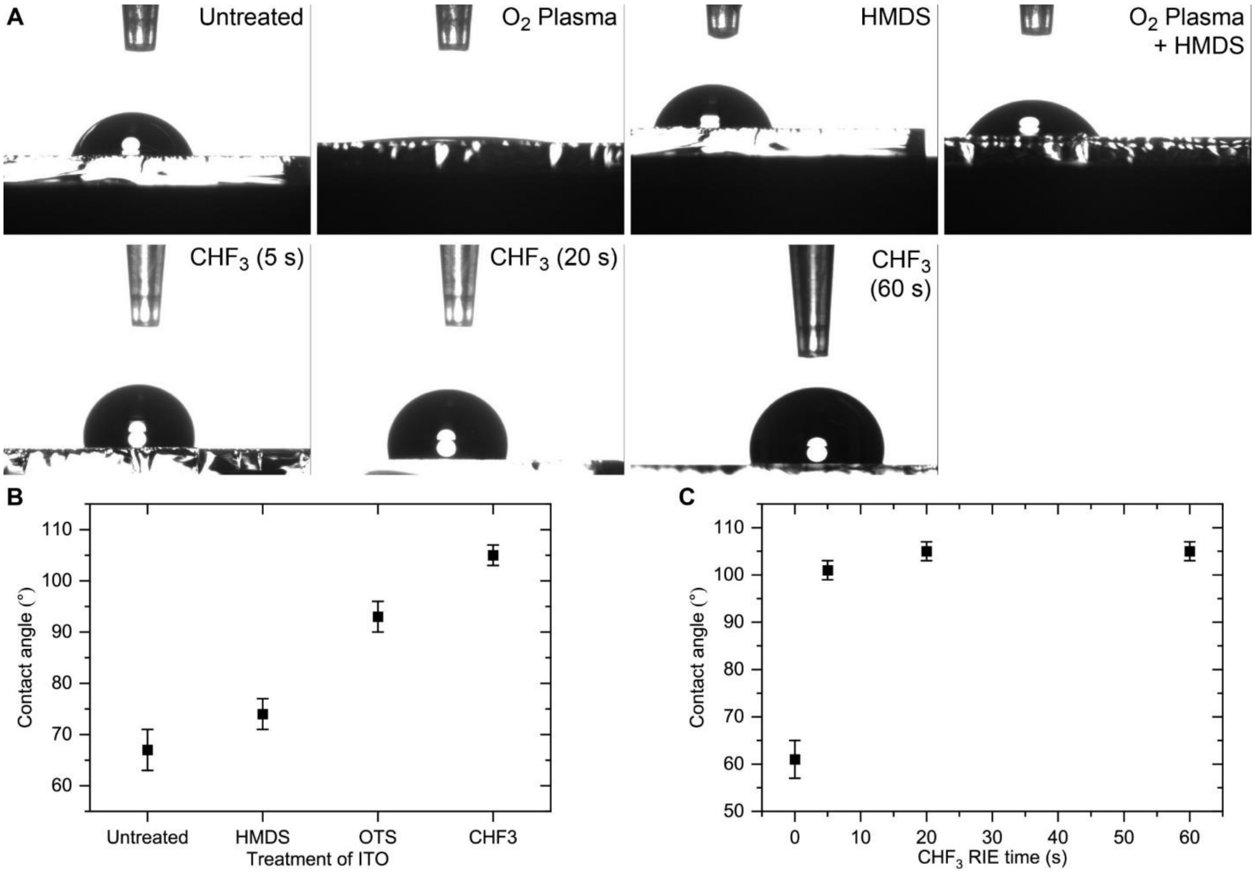

Fig. S18. Contact angle measurement of water on ITO-coated glass after different surface treatments. (A) Optical images of a water droplet on ITO with different surface treatments. (B) Contact angle values for different surface treatments. (C) Water contact angle as a function of reactive ion etching (RIE) time in an CHF_3 atmosphere which functionalized the ITO-coated glass with fluoropolymers.

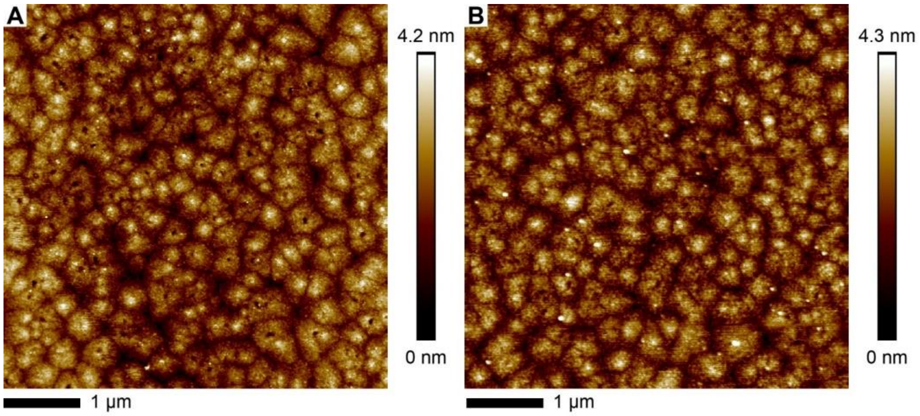

Fig. S19. Effect of fluoropolymer functionalization on the surface roughness of ITO-coated glass characterized by AFM. (A) Pristine substrate (R_q = 0.60 nm). (B) Substrate functionalized with fluoropolymers (R_q = 0.64 nm). Surface roughness was calculated from scans acquired under identical conditions (512 × 512 pixels over a 5 × 5 µm area).

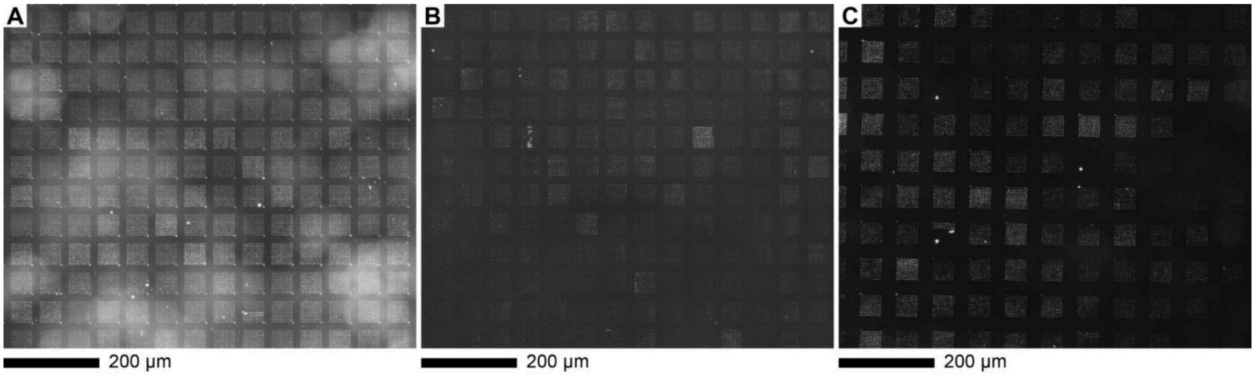

Fig. S20. Large-area organic-inorganic halide perovskite nanocrystal arrays on fluoropolymer-modified ITO-coated glass characterized by dark-field imaging: (A) $MAPbI_3$, (B) $MAPbBr_3$, and (C) $MAPbCl_3$.

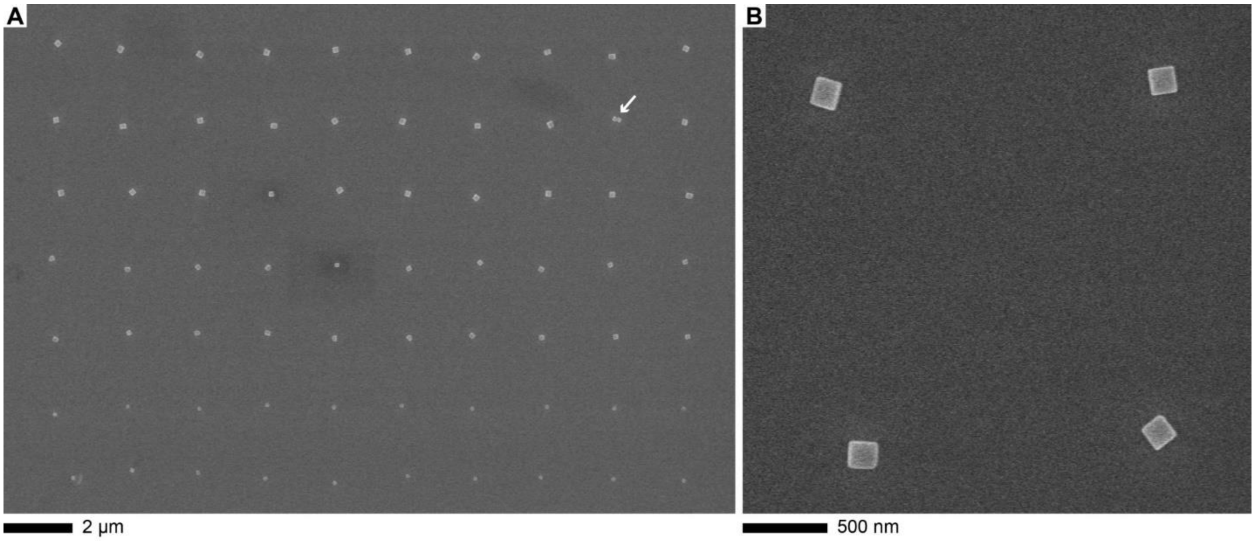

Fig. S21. Size-gradient $MAPbCl_3$ nanocrystal arrays synthesized on fluoropolymer-modified ITO-coated glass. (A) SEM image showing an array with decreasing crystal size from top to bottom. The white arrow indicates a rare case when two nucleation centers emerged during the crystallization. (B) Higher-magnification SEM image showing the rectangular shape of the nanocrystals. Interparticle spacing is ~2 µm.

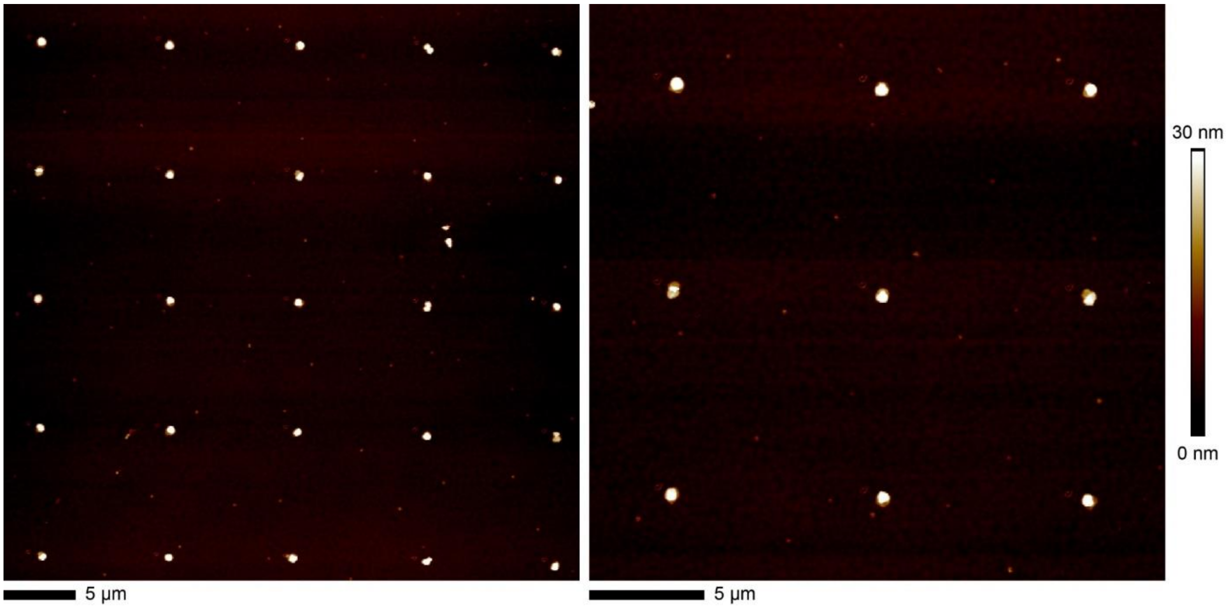

Fig. S22. AFM height images of layered RP-$(BA)_2PbBr_4$ nanocrystal arrays synthesized on fluoropolymer-modified ITO-coated glass.

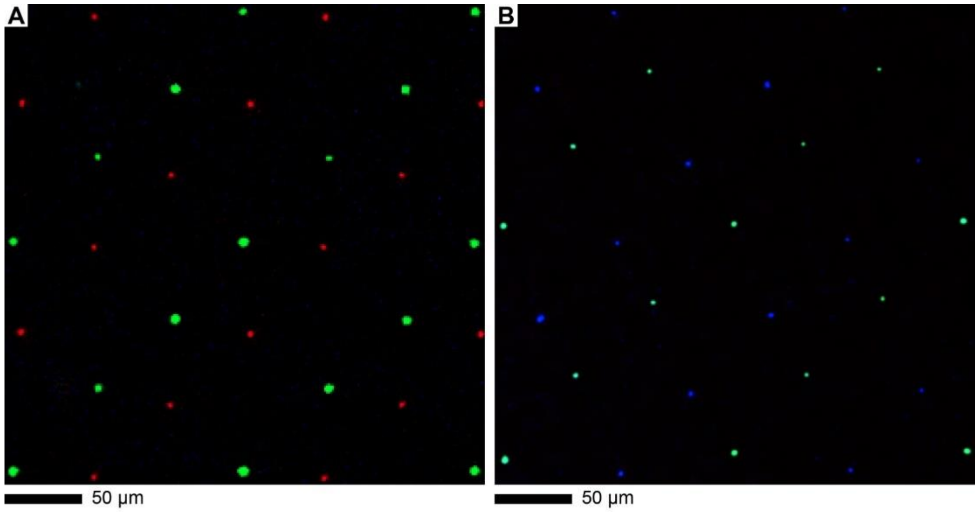

Fig. S23. Merged-channel confocal fluorescence micrographs of two-color halide perovskite nanocrystal arrays comprised of $MAPbI_3$ (red), $MAPbBr_3$ (green), and $MAPb(Br_{0.4}Cl_{0.6})_3$ (blue): (A) red-green array, and (B) green-blue array.

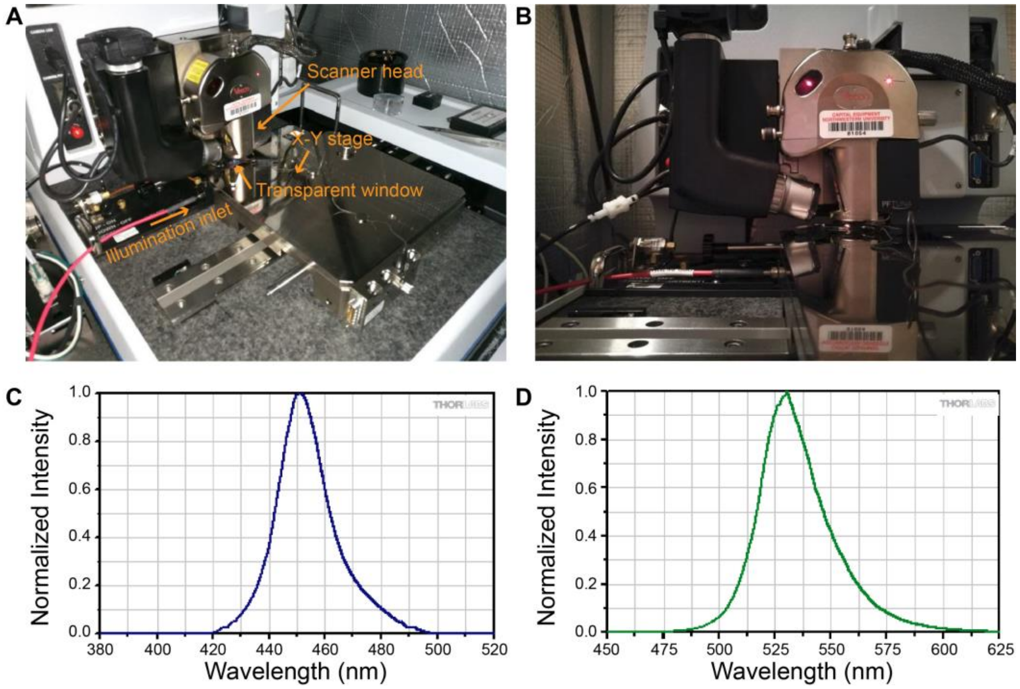

Fig. S24. Setup of the light-illuminated contact-mode AFM test on single-nanocrystal photovoltaics. (A and B) Photos of the AFM setup, where the stage of the AFM has been modified to include light-emitting diodes (LEDs) and a glass window. (C and D) Intensity profiles of the nominally 455 nm (C) and 530 nm (D) LEDs. Photo Credit: Chiara Musumeci, Northwestern University.

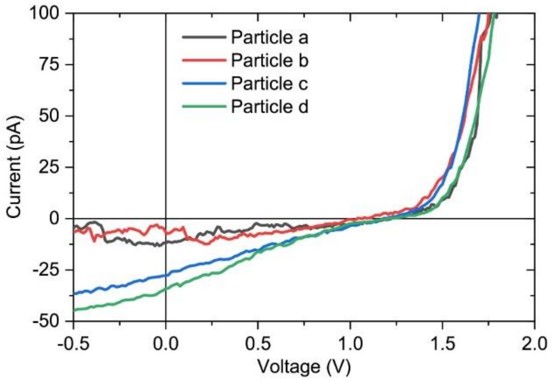

Fig. S25. Current−voltage curves (backward scans) for four individual $MAPbBr_3$ nanocrystals. All curves were obtained under illumination by a 455 nm LED light of ~3.6 mW/cm^2. The curve for particle d is the same one shown in Fig. 6B.

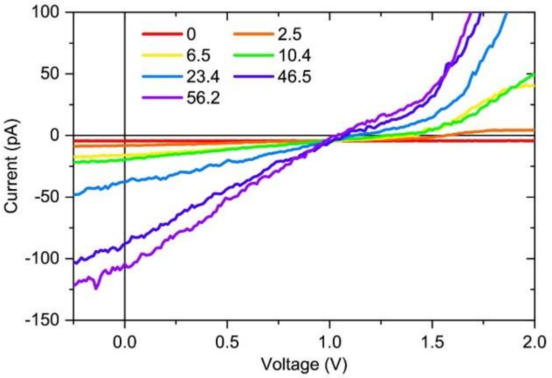

Fig. S26. Light intensity-dependent photovoltaic response from a $MAPbBr_3$ nanocrystal (as in Fig. 6C; backward scans). Light intensity unit: mW/cm^2. Current variation between measurements is mainly attributed to the unstable point contact between the AFM probe and nanocrystal. LED wavelength: 530 nm.

Supplementary Tables

Table S1. Open-circuit voltage (V_{OC}), short-circuit current (I_{SC}), and fill factor (FF) for the four individual $MAPbBr_3$ nanocrystals in Fig. S25. All data obtained under illumination by a 455 nm LED light of ~3.6 mW/cm^2.

**Particle**	**V_{OC} (V)**	**I_{SC} (pA)**	**FF**
Particle a	1.21	11.95	0.23
Particle b	1.06	6.72	0.56
Particle c	1.17	27.80	0.23
Particle d	1.20	34.14	0.20
